# Semiochemicals for intraspecific communication of the fig weevil *Aclees* sp. cf. *foveatus* (Coleoptera: Curculionidae): a first survey

**DOI:** 10.1038/s41598-020-58004-8

**Published:** 2020-01-23

**Authors:** Immacolata Iovinella, Erika Carla Pierattini, Stefano Bedini, Francesca Romana Dani, Salvatore Guarino, Andrea Lucchi, Paolo Giannotti, Giorgio Cuzzupoli, Jessica Girardi, Barbara Conti

**Affiliations:** 1 Department of Biology, University of Florence, Via Madonna del Piano 6, 50019 Sesto Fiorentino, Italy; 2 Department of Agriculture, Food and Environment, University of Pisa, Via del Borghetto 80, 56126 Pisa, Italy; 3 Institute of Biosciences and Bioresources (IBBR), National Research Council of Italy (CNR), Corso Calatafimi 414, 90129 Palermo, Italy

**Keywords:** Animal behaviour, Entomology, Bioanalytical chemistry

## Abstract

The fig tree weevil *Aclees* sp. cf. *foveatus* (Coleoptera: Curculionidae), introduced in Italy in 2005, is currently causing significant economic and environmental losses to fig tree nurseries and orchards in Central Italy. Fig damages are due to the adults feeding on leaves and fruits, and to the galleries dug by the xylophagous larvae in the trunk, which lead the plants to death. To date, no chemical or biological control methods resulted to be effective against this invasive pest. In order to gain information about possible semiochemicals involved in mate recognition and choice, both the volatile organic compounds (VOCs) and the epicuticular lipids of male and female specimens were analysed. VOCs emissions of specimens were characterized essentially by monoterpenes, while epicuticular lipids contained long chained 2-ketones, alkanes, alkenes, including some methyl alkenes, and several fatty acid propyl esters. The attractiveness of reconstituted VOCs blends of the two sexes was tested in electrophysiological and behavioural assays in laboratory conditions. Both the male and the female reconstituted VOCs drove a significant response towards individuals of the opposite sex, thus demonstrating features of sexual attractants. Our results suggest a possible application of VOCs blends as pheromonic attractants in field monitoring and mass trapping of *Aclees* sp. cf. *foveatus*.

## Introduction

Fig (*Ficus carica* L., Moraceae) is an important tree crop, widespread in the Mediterranean basin, with Turkey being the main producer of fresh figs with 305,700 t, followed by Egypt (177,100 t), Morocco (137,900 t), Algeria (128,700 t), Spain (36,000 t), Tunisia (22,500 t), Albania (20,000 t), and Italy (11,300 t)^1^.

In Italy, fig nurseries and orchards are facing in recent years severe damages related to the presence of an exotic weevil pest belonging to the genus *Aclees* (Coleoptera: Curculionidae), which has significantly reduced the fig production. In fact, since its introduction^2^ due to the trading of *Ficus* spp. plants for ornamental purposes^3^, Italian fresh fig production has decreased from 20,091 t in 2015 to 11,363 t in 2017, and the cultivated area has respectively decreased from 3,478 ha to 2,336 ha^1^.

Plant damage is due to adult feeding on leaves and early stage fruits but mostly to the feeding behaviour of the larvae, which live as xylophagous in all the woody parts of the plant, causing the complete destruction of xylem and phloem, so leading the plant to death^4^. This pest, initially identified as *A. cribratus* Gyllenhal 1835^2^, has been subsequently found to have closer morphological similarity with *A. foveatus* Voss 1932^5^. However, a specific attribution of this weevil is still to be confirmed.

To date, no chemical or biological control strategies have been able to prevent the *A*. sp. cf. *foveatus* spreading^6,7^. As for other Coleoptera^8^, mass trapping technique seems to be the only possible solution to manage this invasive pest^9^. On the contrary, treatments against the larval stages are nearly unfeasible since they live protected inside the dug tunnels. Traps associated with aggregation or sexual pheromones have been demonstrated to be highly effective to control Curculionidae beetles such as *Rhynchophorus palmarum* (Linnaeus)^10^, *R. ferrugineus* Oliver^11–13^, *Rhinostomus barbirostris* (Fabricius)^14^, and *Araecerus fasciculatus* (De Geer)^15^.

The aim of the present study was to investigate and characterize the semiochemicals involved in intraspecific communication of *A*. sp. cf. *foveatus*, to establish a possible pheromonic nature of these compounds, and to lay the foundations for the development of an attractant source that could be exploited for monitoring and mass trapping purposes in an integrated pest management (IPM) perspective.

## Materials and Methods

### Insects rearing

Adults of *A*. sp. cf. *foveatus* were collected in a fig orchard (Carmignano, PO, Italy, 43°48′36.97″N 11°00′53.78″E) both in late spring (May) and late summer (September) and maintained into 75 × 75 × 115 cm cages (Bug-Dorm-2400 Insect Rearing Tent, MegaView Science Co. Ltd., Taiwan) under laboratory conditions (25 ± 1 °C, 65% relative humidity, natural photoperiod) providing them with fig branches and fruits that were renewed every two days, and water *ad libitum*.

### Chemical analysis

Specimens for VOC analyses were sacrificed by freezing at −20 °C. Since in the Palm Weevil *Rhynchophorus palmarum* L. (Coleoptera: Curculionidae) male pheromones are more concentrated in the prothorax and in the rostrum^6^, heads and pro-thoraxes of *Aclees* sp. cf. *foveatus* were dissected and stored at −20 °C for the volatile analyses. The fertility of the sacrificed specimens was evaluated through dissection as well. The female fertility was evaluated by assessing the presence of eggs in the ovarioles while the fertility of the males was evaluated by assessing the size of testes and associated glands.

Since field observations about mating rituals of *A*. sp. cf. *foveatus* have underlined that rostral rubbing by males on the female body in the pre-copulatory phase is a key factor for the success of the copulatory attempt^7^, we hypothesized that head and pro-thorax could be the putative sites of sexual pheromone production. To this aim, headspace analyses have been carried out on three pools of heads plus pro-thoraxes and three pools of meso-thoraxes and meta-thoraxes plus abdomens, each obtained from three males and three females separately, for a total of nine males and nine females collected in May. The same number of samples was analysed for specimens collected in September.

Samples were inserted into 2 mL vials (Supelco) for 20 min at 30 °C and volatiles were absorbed with a Divinylbenzene, Carboxen, Polydimethylsiloxane (DVB/CAR/PDMS; 1 cm, 30–50 µm thickness) SPME fibre. SPME extraction of empty vials, of the insect frass and of fig fruits and branches used for the insect rearing were also performed in order to factor out VOCs contaminants.

Analyses were carried out on a GC-MS 7820 GC system-5977B MSD (single quadrupole, Agilent Technologies) equipped with a 7693 autosampler (Agilent Technologies). SPME fibres were desorbed in the injection chamber for 10 min. The separation was carried out using a 19091S-433UI column (stationary phase, 95% PDMS, 5% benzene; 30 m × 0.25 mm, Agilent Technologies), using helium as carrier gas (1 mL min^−1^). Oven temperature gradient as follows: 45 °C (2 min); 10 °C min^−1^ up to 200 °C (3 min); 15 °C min^−1^ up to 300 °C (2 min). Electronic ionization was carried out at 70 V and acquired m/z ranged from 50 to 550. A mixture of linear alkanes (C_15_-C_30_) was injected under the same conditions and used as reference to calculate retention indexes of sample analytes.

Data were analysed using Agilent MassHunter Qualitative Analysis B.07.00 software. Compounds were identified by spectra comparison with Wiley275 and NIST11 libraries, as well as by comparing their retention indexes with those reported in spectral libraries. Identification of compounds was confirmed by injection of standards (all purchased from Sigma-Aldrich) under the same analytical conditions. The relative abundance of each compound exclusive to *Aclees* sp. cf. *foveatus* samples was calculated as the percentage of the area underlying the peak total ion chromatogram (TIC) with respect to the sum of the peak area of all compounds exclusive to insect samples. On the basis of such results, the volatile blends of males and females were reconstituted using the commercial standards of the most abundant compounds identified in the headspace analyses. The reconstituted male VOCs (weight/total weight) was composed by limonene (31%), α-pinene (18%), α-phellandrene (11.4%), β-myrcene (6%), β-pinene (5%), sabinene (3.5%), camphene (0.8%) and hexane (24.3%) as solvent. The reconstituted female VOCs was composed by limonene (41.6%), decanal (2.4%) α-phellandrene (2.4%), β-myrcene (2.15%) and hexane (48.55%) as solvent.

Since males have been suggested to select mating partners on the basis of cuticular cues assessed through the rubbing behaviour^7^, we also analysed the cuticle lipids of females and males collected in May and September. For each sample, three pools of the heads plus pro-thoraxes of three specimens were extracted in 1 mL of pentane at room temperature for 5 minutes. Extracts were then evaporated under a gentle nitrogen line stream and then resuspended in 25 µl of heptane. 3 µl of the solution were injected in the same GC-MS equipment described above using the same chromatographic column. Helium (1 mL min^−1^) was used as carrier gas. Oven temperature program was set as follows: 70 °C (3 min); 15 °C min^−1^ up to 150 °C (3 min); 5 °C min^−1^ up to 320 °C (17 min). Electronic ionization was carried out at 70 V and acquired m/z ranged 50–650.

A mixture of linear alkanes (C_21_-C_43_) was injected under the same analytical conditions to calculate the retention indexes of target analytes. Data were analysed using the software Agilent MassHunter Qualitative Analysis B.07.00. Compounds present in the extracts were identified using mass spectral libraries as reported above and for cuticular hydrocarbons through manual identification of spectra and comparison with those reported in literature^16–20^. The relative abundance of each compound was calculated as the percentage of the area underlying the peak total ion chromatogram (TIC) with respect to the sum of the area of the peaks present.

### Electroantennography

The reconstituted volatile blends of both sexes were tested against males and females of *A*. sp. cf. *foveatus* by Electroantennogram techniques (EAG) using the same protocol by Guarino *et al*.^21^, with some modifications. Experiments were conducted using 1 μL of each reconstructed blend extract or a solvent (hexane) as control, that was loaded on a filter paper section (Whatman, grade 1) of 2.0 cm^2^. The stimulus was exposed to the air for 30 s for solvent evaporation and then placed inside a glass Pasteur pipette. Puff stimuli were blown into an airflow that passed over the antennal preparation using a stream controller (model CS-05; Syntech, Hilversum, the Netherlands) to generate a 1.5 s stimulus at 1 min distances, with a flow rate of 1.5 l min^−1^. The signals elicited on the antennae were handed through a high-impedance amplifier (model IDAC-4, Syntech) and recorded with specific software (Syntech). For the EAG preparations, adult *A*. sp. cf. *foveatus* were anesthetized by cooling them at about − 4 °C for 40 s, the antenna was cut and mounted on the reference electrode made by a glass capillary tube (1.5 mm diameter) filled with 0.1 M KCl solution and connected with silver wire to the amplifier. A similar capillary glass electrode was used as recording electrode and placed in order to touch gently the tip of the antenna. The capillary tubes were prepared using a microelectrode puller (Narishige PC-10, Tokyo, Japan) to achieve an appropriate diameter that enabled the insertion onto the antennal tip. A total of ten insects (sex ratio 1:1) were used for the EAG bioassays.

### Behavioural assays

The behaviour of *A*. sp. cf. *foveatus* adults in the presence of the reconstituted VOCs was evaluated separately for males and females, using a two-choice bioassay conducted in a steel arena (32 cm ∅ × 12 cm height) with two diametrically opposed holes (3 cm ∅) on the bottom located 3 cm from the sidewall^22^. For each replicate, 2 µl of ethanol (control) and a 0.01% reconstituted VOCs blend (diluted using ethanol) were applied on a filter paper disk (1 cm ∅), suspended at the centre of each hole by a cotton thread taped to the lower surface of the arena; 500 mL glass flasks with paraffin oil-coated neck were positioned under each hole. This was done because preliminary trials demonstrated that paraffin oil has neither repellent nor attractant effect on insects, and prevent their return onto the arena, once they have made a choice.

For each replicate, six insects (males or females, separately) were placed in the centre of the arena under a clean glass cup (180 mL) and allowed to acclimate for 30 min. Then, the glass flask was removed, and the arena was covered with a steel lid and left for 24 h in the dark at 25 ± 1 °C and 65% relative humidity. After each trial, the arena was carefully washed with soap and rinsed throughout with tap water and ethanol. Twelve replicates were performed for each assay, for a total of 72 females plus 72 males tested for the reconstituted male emission and 72 females plus 72 males for the female emission; each individual did not undergo more than one trial.

### Statistical analysis

Differences in the compound relative abundance of VOCs in head plus pro-thorax samples of females and males collected during late spring were analysed by Mann-Whitney U test. The abundance of the epicuticular lipid components were compared between sexes (by Mann-Whitney U test) by considering both the specimens collected in May and those collected in September. Similarly, we compared epicuticular lipid components of specimens collected in these two periods independently by their sex. In both cases 0.025 was considered as probability threshold.

Mean values of the antennal depolarization responses elicited by the various test stimuli in EAG trials with *A*. sp. cf. *foveatus* antennae were analysed by one-way ANOVA followed by Fisher LSD’s test.

Data obtained from the behavioural assay were analysed by likelihood chi-square test, with a null hypothesis of a 50:50 chance of insects choosing the control versus the VOCs-treated chamber. The numbers of non-choosing insects were analysed by one-way ANOVA and multiple comparisons performed by orthogonal contrasts. All the statistical analyses were performed using IBM SPSS Statistics version 22.0 software.

### Ethical approval

All applicable international, national, and/or institutional guidelines for the care and use of animals were followed.

### Informed consent

Informed consent was obtained from all individual participants included in the study.

## Results

### Composition of VOCs and epicuticular lipids

**Figure 1:**
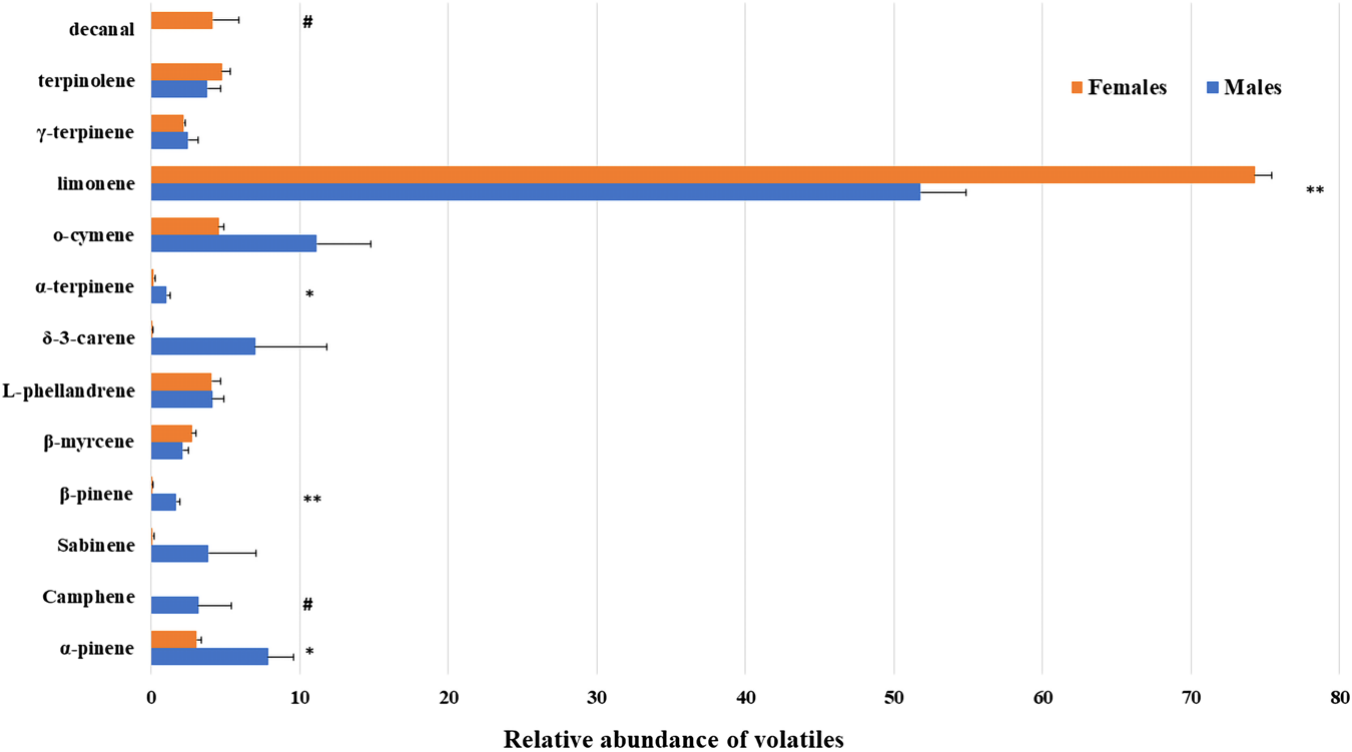
Relative abundance of volatile compounds in head plus pro-thorax samples of *Aclees* sp. cf. *foveatus* males and females collected in May. ^#^compound exclusive of one sex. Asterisks indicate significant differences (Mann-Whitney U test, n = 5; *P ≤ 0.05; **P ≤ 0.01). Bars represent standard error.

The head plus pro-thorax samples of both females and males collected in May showed a rich volatile profile, though the male blend was more intense (about 100 times) and diverse for its components. Both for males and females, all the compounds present in the insect samples but not in control samples (vegetal material and frass) were identified. Although limonene is the component with the highest relative abundance in both sexes, it is more concentrated in female samples than in males, where it coelutes with traces of eucalyptol. In females, terpinolene and o-cymene represent the second more abundant compounds, differently from males where o-cymene, α-pinene, and δ-3-carene are the most abundant components after limonene. Decanal was found to be exclusive for females, while camphene was found exclusively in male extracts. Several compounds were found to be significantly different in their relative amount between sexes (Fig. 1). Pools of meso-thoraxes, meta-thoraxes and abdomens showed a similar volatile profile (in terms of identified volatiles and relative abundance) as heads and pro-thoraxes.

Samples prepared from adults collected in September contained only a few VOCs and in low abundance. Most of these components were also present in control samples and no evident differences between sexes were observed. Camphene was the only compound of the male samples identified both in May and September specimens, while only decanal was present in the females of both periods. Traces of longiciclene were found only in September male specimens.

Such remarkable differences between May and September specimens could be linked to fertility. Females sacrificed in May were found to have very developed ovaries, while those collected in September had either much less developed or undeveloped ovarioles. A similar situation was found in males where testes and associated glands were much more developed in specimens collected in May.

**Figure 2:**
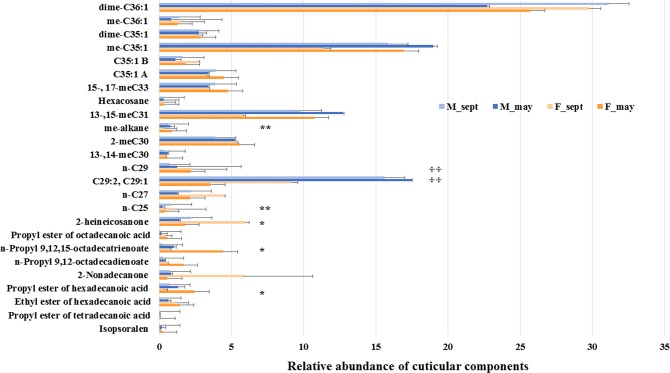
Relative abundance of epicuticular lipid components in head plus pro-thorax of *Aclees* sp. cf. *foveatus* males and females collected in May and September. Asterisks indicate significant differences between seasons (May, September) (Mann-Whitney U test, n = 5; ^*^P ≤ 0.025; ^**^P ≤ 0.01). Crosses indicate significant differences between sexes. (Mann-Whitney U test, n = 5; ^**✢**^*P* ≤ 0.025; ^**✢✢**^*P* ≤ 0.01). Bars represent standard error.

All the epicuticular extracts contained a mixture of long-chain paraffines and olefins, long chained 2-ketones, propyl esters of fatty acids (Fig. 2), and free fatty acids. These latter compounds are ubiquitarians in insect specimens extracted in apolar organic solvents and probably derive from internal soft tissues.

Cuticular hydrocarbon mixture contained, besides linear and methyl branched alkanes and linear monoenes and dienes, several branched alkenes which are uncommon components of insect cuticular lipids^18–20^. Their methyl branched structure was compatible with their molecular ions, with the presence of even mass ions in the interval 182–322 m/z and with the retention indexes^18,19^. Only a few mass spectra of methyl branched long chained alkenes are reported in the literature, and to the best of our knowledge general rules about the interpretation of their spectra are only been reported by Brown *et al*.^19^. Since the unsaturation position was not determined in the current work the IUPAC name could not be assigned.

No one of the identified epicuticular compounds were found to be exclusive of females or males; however, the mixture of nonacosene and nonacosadiene was more abundant in males than in females (Mann-Whitney U test, *P* < 0.004), while *n*-nonacosane was more abundant in females (Mann-Whitney U test, *P* < 0.004). By considering specimens collected in May and September, independently by their sex, we found 5 compounds to be significantly different in their relative abundance (Fig. 2).

### Electroantennography

**Figure 3:**
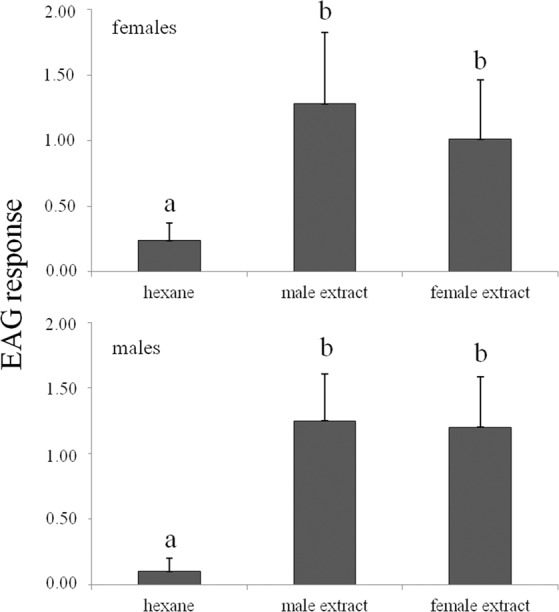
Electroantennogram responses (mV) (mean + SE) of *Aclees* sp. cf. *foveatus* males and females (n = 10). Different letters indicate that values differ statistically at *P* ≤ 0.05; one-way ANOVA followed by LSD’s test.

The results of the EAG bioassays carried out on *A*. sp. cf. *foveatus* males and females are reported in Fig. 3. The reconstituted blends of volatile organic compounds identified in the extracts of females and males elicited significantly higher EAG responses than the solvent used as control, both in *A*. sp. cf. *foveatus* male (*F* = 9.35; df = 2; *P* < 0.01) and female antennae (*F* = 5.44; df = 2, *P* < 0.05).

### Behavioural assays

**Figure 4:**
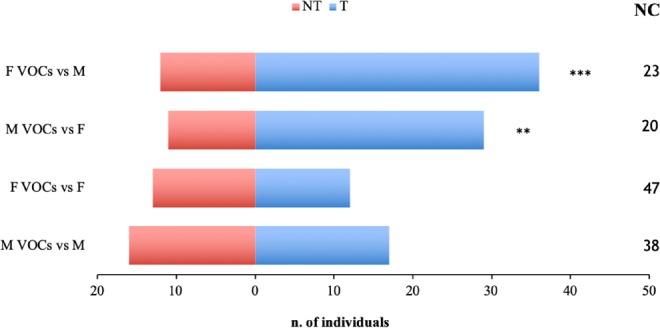
Behaviour of adults of *Aclees* sp. cf. *foveatus* in the presence of reconstituted volatile organic compounds (VOCs). Histograms represent the number of insects that choose the cue. F VOCs *vs* M, female VOCs *vs* males; M VOCs *vs* F, male VOCs *vs* females; F VOCs *vs* F, female VOCs *vs* females; M VOCs *vs* M, male VOCs *vs* males. T, number of insects trapped in the flask treated with the reconstituted VOCs; NT, number of insects trapped in the flask not treated; NC, number of non-choosing individuals. Asterisks indicate significant differences in the number of the choosing insects (*χ*^2^ test; **P ≤ 0.01; ***P ≤ 0.001).

The two-choice pitfall assays carried out on *A*. sp. cf. *foveatus* males and females separately evidenced that the reconstituted VOCs have no effects on same-sex adults, while they resulted attractive for the opposite sex (Fig. 4). In particular, the female VOCs resulted highly effective on males (*χ*^2^ = 12.00; n = 72; *P* = 0.001) (Fig. 4). In line with the attractiveness results, the mean number of individuals that did not make a choice varied from 1.67 ± 1.07 to 3.93 ± 1.38, depending on the sex of the insects and the VOCs with differences statistically significant (*F* = 8.23; df = 3; *P* < 0.001). In particular, orthogonal contrast test showed that the number of non-choosing insects was significantly higher when insects were exposed to reconstituted VOCs of the same-sex than those exposed to the opposite sex VOCs (same-sex VOCs *vs* opposite sex VOCs, *t* = −4.74; df = 44; *P* < 0.001).

## Discussion

Males and females of *A*. sp. cf. *foveatus* were found to differ both for the volatile emission and for their epicuticular lipids; however, while for these latter the differences were merely due to the relative amounts of their components, a few sex-specific compounds were found in the volatile mixtures.

Some of the compounds identified in the epicuticular extracts are quite new and uncommon for insects. In fact, methyl branched alkenes have been reported only for a few species scattered between different orders^18–20,22–24^, and among Coleoptera, to the best of our knowledge, they are only known for two longicorn beetles^18,25^. Similarly, n-propyl esters of fatty acids, are uncommon in cuticular lipids^16,26^. Behavioural assays are needed to test if cuticular lipids profile, which differs between sexes, is the proximate cue assessed through rostral rubbing by males before mating^5^.

While VOCs do not require proximity to be sensed, cues entailed within the cuticular waxes require contact between potential sexual partners. The behaviour of rostral rubbing and antennal tapping performed by males on the body of potential partners^5^, may be functional to assess sex-specific profiles of epicuticular lipids and, possibly, the fertility of mates. Similar behaviour has also been described in other Coleoptera species and considered to be linked to short-range partner recognition^27,28^.

All the compounds observed in the insect VOCs, with the exception of decanal, are monoterpenes. The presence of several compounds in common between males and females (limonene, terpinolene, L-phellandrene, o-cymene, γ-terpinene, β-myrcene, and α-pinene) corroborates the hypothesis that homosexual interactions, typical in this species, can be promoted by a similar volatile emission^7^. Electroantennogram experiments revealed that both sexes perceive the VOCs from both extracts indicating the responsiveness of the *A*. sp. cf. *foveatus* adults to monoterpenes as observed in other curculionid species as *R. ferrugineus*^29^. However, the differences in the relative abundance and the few compounds exclusive of the VOCs in the two sexes could be responsible for the clear sex-specific attractiveness observed in the bioassays. Moreover, the remarkable differences observed between the compound-reach VOCs of the May specimens and the faint profile of insects collected in September could be linked to fertility. In fact, females sacrificed in May were found to have very developed ovaries and eggs, while those collected in September had either much less developed or undeveloped ovarioles and no eggs. A similar situation was found in males, where testes and associated glands were about twice in size in May specimens with respect to September males (Data not showed).

All the monoterpenes identified in the VOCs are commonly found in several plant extracts, and some have been reported to show attractant properties towards Curculionidae beetles, such as *Dendroctonus* spp.^30,31^. Presence of plant monoterpenes as the main component of insect VOCs may be due to sequestration of plant secondary compounds acquired through a phytophagous diet^32^. If this was the case in *A*. sp. cf. *foveatus*, given the identical rearing conditions used for all specimens during our experiment, different sequestration and storage of plant monoterpenes are likely to occur in the two sexes, finally leading to gender-specific VOC profiles. However, it is important to notice that the monoterpenes we reported as insect VOCs were not present in the SPME extracts (data not showed) of the fruits and twigs we used for the insect rearing and therefore if the VOCs are acquired through the diet, this possibly occurs through accumulation of low abundance compounds present in the adult diet or during the larval stage when insects feed on the plant wood. Studies on the volatiles from *F. carica* have also found absence or paucity of monoterpenes. A study aimed at identifying volatiles released by receptive and unreceptive figs attracting the species-specific pollinator wasp *Blastophaga psenes* (L.), failed to identify monoterpenes in the pentane extracts^33^. SPME-sampled volatiles from Portuguese *F. carica* varieties, contained limonene, the main compound we identified in the insect VOCs, only in low relative abundance both in leaves and fruits, and traces of α- and β-pinene and of eucalyptol only in the fruits^34^. Leaves and fruits of different Tunisian fig cultivars extracted through hydrodistillation were found to differ for the presence of monoterpenes, with only α-pinene being constantly present, δ-3-carene being identified in few samples and traces of limonene being present in the leaves of only one variety^35^. Given such differences, further studies are needed to understand if feeding on different fig cultivar affects the composition of *A*. sp. cf. *foveatus* volatile blend.

Chemical communication based exclusively on plant terpenes has been found in the raspberry weevil *Aegorhinus superciliosus* (Guérin-Méneville) where, differently from males which release only α-pinene, females release α-pinene and limonene and this blend was found to attract males both in laboratory bioassays and in the field^36^.

Beside releasing died-acquired secondary compounds, several Curculionidae synthetize monoterpene-derivatives by converting plant terpenes. This condition has been demonstrated for grandisol, the main component of the sex pheromone of the cotton boll weevil (*Anthonomus grandis* Boheman)^37^, and for verbenol, produced both by *Ips pini* (Say) and by species of the genus *Dendroctonus*^38^. In addition, *de-novo* biosynthesis of the oxygenated terpenoid ipsdienol, one of the components of *I. pini* aggregation pheromone, has also been reported^32,39^. In other cases, volatiles unrelated to terpenes have been identified as semiochemicals, as for example several branched secondary alcohols reported for the genus *Rhynchophorus*^13^, and brevicomin, found in species of the genus *Dendroctonus*^40^. These cases do not occur in *A*. sp. cf. *foveatus*, where except for decanal we only identified terpenes.

The bioassays we conducted under laboratory conditions have demonstrated that the reconstituted VOCs of both females and male attract the opposite sex and do not trigger a sex-aspecific aggregation behaviour. Since the biology of the species has not been fully described yet, it is uncertain if, as it occurs in other xylophagous curculionids^32,41^, aggregation of adult individuals occurs and if it is contextual to courtship and mating. In any case, our results suggest that aggregation and mating are possibly mediated by different semiochemicals.

Though our observations suggest a possible role for sex-specific VOC blends to be used for future monitoring of field populations of *A*. sp. cf. *foveatus*, as well as for mass trapping in an IPM perspective, further studies are needed to test the effect of the identified blends under natural conditions with the aim to help developing control strategies against this invasive pest.
